# Bacterial community assembly in Atlantic cod larvae (*Gadus morhua*): contributions of ecological processes and metacommunity structure

**DOI:** 10.1093/femsec/fiaa163

**Published:** 2020-08-20

**Authors:** Ragnhild I Vestrum, Kari J K Attramadal, Olav Vadstein, Madeleine Stenshorne Gundersen, Ingrid Bakke

**Affiliations:** Department of Biotechnology and Food Science, NTNU - Norwegian University of Science and Technology, Trondheim, Norway; Department of Biotechnology and Food Science, NTNU - Norwegian University of Science and Technology, Trondheim, Norway; Department of Biotechnology and Food Science, NTNU - Norwegian University of Science and Technology, Trondheim, Norway; Department of Biotechnology and Food Science, NTNU - Norwegian University of Science and Technology, Trondheim, Norway; Department of Biotechnology and Food Science, NTNU - Norwegian University of Science and Technology, Trondheim, Norway

**Keywords:** microbiota, microbial ecology, community assembly, Atlantic cod, metacommunity, ecological processes

## Abstract

Many studies demonstrate the importance of the commensal microbiomes to animal health and development. However, the initial community assembly process is poorly understood. It is unclear to what extent the hosts select for their commensal microbiota, whether stochastic processes contribute, and how environmental conditions affect the community assembly. We investigated community assembly in Atlantic cod larvae exposed to distinct microbial metacommunities. We aimed to quantify ecological processes influencing community assembly in cod larvae and to elucidate the complex relationship between the bacteria of the environment and the fish. Selection within the fish was the major determinant for community assembly, but drift resulted in inter-individual variation. The environmental bacterial communities were highly dissimilar from those associated with the fish. Still, differences in the environmental bacterial communities strongly influenced the fish communities. The most striking difference was an excessive dominance of a single OTU (*Arcobacter*) for larvae reared in two of the three systems. These larvae were exposed to environments with higher fractions of opportunistic bacteria, and we hypothesise that detrimental host–microbe interactions might have made the fish susceptible to *Arcobacter* colonisation. Despite strong selection within the host, this points to a possibility to steer the metacommunity towards mutualistic host–microbe interactions and improved fish health and survival.

## Teaser

Bacterial community assembly in Atlantic cod larvae is driven by deterministic and stochastic processes and influenced by the bacterial metacommunity structure.

## INTRODUCTION

There has been a great advancement in our understanding of the microbiota associated with animal hosts and its roles in host health and development. The gut microbiota plays important roles in epithelial differentiation and maturation (Naito *et al*. 2017), contributes to metabolism of nutrients and xenobiotics (Semova *et al*. 2012; Sonnenburg and Bäckhed 2016; Koppel, Maini Rekdal and Balskus 2017) and is essential for the development of the immune system (Hiippala *et al*. 2018). The indigenous microbiota also protects the host by preventing colonisation by harmful bacteria (Lazado *et al*. 2011; Lazado and Caipang 2014; Hiippala *et al*. 2018). Dysbiosis in the gut microbiota is associated with an increasing number of diseases (Rogers *et al*. 2016; Brugman *et al*. 2018), and the microbiota can affect both growth and survival (Vadstein *et al*. 2018). Consequently, there is great interest in understanding factors and processes that determine the composition of the animal gut microbiota, which have been proposed to include host genetics, developmental stage (Bonder *et al*. 2016), diet (David *et al*. 2013), environmental microbes (Fujimura *et al*. 2014) and selection in the host (Rawls *et al*. 2006). However, in natural habitats these factors are often interacting, and are thus hard to study. For example, it was only recently revealed that host genetics has a relatively small impact on the gut microbiota of humans compared to the impact of environmental factors such as geographical location, diet and age (Jackson *et al*. 2018; Rothschild *et al*. 2018).

Fish larvae are well suited for experimental studies of community assembly and dynamics of vertebrate-associated microbiota, because of their small size, rapid development, possibilities for good sample size and replication, and a wide range of experimental systems and host species (Vestrum *et al*. 2018). During the early colonisation of skin and intestinal system of fish the major source for bacteria entering the fish is assumed to be the surrounding water microbiota (Nayak 2010). The gut is colonised at the mouth opening in young marine fish larvae (Reitan, Natvik and Vadstein 1998). We have previously shown that it is possible to optimise microbial water quality and promote mutualistic host–microbe interactions for cultivated fish by applying ecological theory to set up selection regimes through water treatment (Vadstein *et al*. 2018). Attramadal *et al*. (2014) demonstrated that optimising microbial water quality in both recirculation aquaculture system (RAS) and a microbially matured flow through system (MMS) lead to a 70–90% increase in the survival of Atlantic cod (*Gadus morhua*) larvae compared to traditional flow through rearing systems (FTS). Moreover, we have also shown that cod larval microbiota is affected by the differences in the water microbiota introduced through water treatment (Vestrum *et al*. 2018). Paradoxically, despite this, the fish microbiota is generally highly dissimilar from microbiota in the surrounding water, indicating that selection in the host structures the fish microbiota (Sullam *et al*. 2012; Bakke *et al*. 2015; Giatsis *et al*. 2015). On the other hand, high inter-individual variations between fish in the same environment (Fjellheim *et al*. 2012; Boutin *et al*. 2014) suggest that also stochastic processes like drift and dispersal contribute to microbial community assembly in the host. In general, the relative importance of the various processes and factors influencing the colonisation of animals and especially fish is poorly understood.

Ecological theory has been proposed as a foundation to increase our understanding of host-associated microbiota. Vellend (2016) suggested that four fundamental ecological processes explain patterns in community diversity and composition: selection, dispersal, speciation and drift. Nemergut *et al*. (2013) argue that speciation should be considered diversification in the case of microbial community assembly, as for microbes the species concept is complicated, and the generation of new genetic variation can bring change to a community's dynamics even if new species are not created. Thus, we choose to use diversification when describing this process. Vellend's conceptual synthesis has been found useful also for microbial community assembly (Hanson *et al*. 2012; Nemergut *et al*. 2013), but few have used it for animal hosts. Two studies on zebrafish by Burns and colleagues (Burns *et al*. 2016; Burns *et al*. 2017) concluded that drift and passive dispersal were sufficient to generate substantial variation in the microbiota across individual hosts, and that interhost dispersal can be more important than differences in host immunity. Dispersal of species has the potential to link local communities into what has been defined as a metacommunity (Leibold *et al*. 2004). Traditional metacommunity theory assumes that local communities occur in different patches that are linked through dispersal. Metacommunity theory explains patterns in community composition as a combination of local factors (selection) and regional factors (dispersal between patches). Thus, the patches can also exhibit heterogeneity or similarity over time and space due to variations in dispersal and selection pressure. Miller *et al*. (2018) have proposed extensions to the traditional metacommunity theory to include host–microbiota systems. The authors argue that dispersal occurs both between hosts and between hosts and the environment, and that feedback between the hosts and the environmental microbiota could influence the host microbiota. In addition they propose that the host-associated microbiota may have the ability to change host properties such as fitness and development (Miller, Svanbäck and Bohannan 2018).

In this study, we examine the bacterial community assembly in newly hatched Atlantic cod larvae over a period of 46 days, through detailed characterisation of the bacterial communities of water, feed and individual fish. The fish were reared in triplicate tanks with water from three distinct source bacteria over a period of 30 days, followed by a period of 16 days where all tanks received water with the same bacterial communities. Until 30 days post hatching (dph), we consider each of the three systems, including water, fish and feed, separate metacommunities. We quantified the relative importance of ecological processes under action and elucidated the relationship between the bacterial communities of the water and the fish.

## MATERIALS AND METHODS

For this study we analysed the bacteria of cod larvae, water and live feed samples originating from a start feeding experiment with cod larvae, previously described in Attramadal *et al*. (2014). The experimental setup is described briefly below, and further details are given in Attramadal *et al*. (2014). The experiment was carried out at NTNU Sealab within the Norwegian animal welfare act guidelines, in accordance with the Animal Welfare Act of 20 December 1974, amended 19 June 2009, at a facility with permission to conduct experiments on fish (code 93) provided by the Norwegian Animal Research Authority (NARA).

### Experimental design and rearing systems

The primary experimental variable in this study was the use of three different water treatments systems to create three distinct microbial communities entering the rearing tanks. These systems were a flow through system (FTS), a microbial maturation system (MMS), and a recirculation aquaculture system (RAS). In FTS and MMS, the carrying capacity (i.e. the maximum cell number that can be sustained over time by the resources available) of the water going into the tanks (incoming water) was significantly lower than in the rearing tank water, while for RAS, it was more or less the same for incoming water and tank water (Attramadal *et al*. 2014; Vadstein *et al*. 2018). Thus, FTS incoming water was considered to represent r-selected microbial communities, and RAS and MMS K-selected microbial communities (Attramadal *et al*. 2014; Vadstein *et al*. 2018). In each system there were three replicate fish rearing tanks (of 160 L) which were maintained from hatching to 30 dph. Thereafter all the nine rearing tanks received the same microbial water quality (i.e. MMS water) until 46 dph. Details about the water treatments in the three systems are found in Attramadal *et al*. (2014).

### Cod larvae rearing

Fertilised Atlantic cod eggs were received from Nofima marine national breeding station, Havbruksstasjonen i TromsøAS. In brief, eggs were disinfected with glutaraldehyde and transferred to the rearing tanks to reach a final density of 100 larvae L^−1^. The larvae were fed rotifers (*Brachionus* ‘Cayman’) from day 3–26 dph, *Artemia* nauplii from day 22–32 dph and formulated feed (GEMMA Micro, SKRETTING, Norway) from day 31–46 dph. More details about the egg handling, cod larvae rearing and calculations of the survival of the cod larvae at 32 dph are found in Attramadal *et al*. (2014) and in Supplementary Table S1, see online supplementary material. The survival of the cod larvae was not calculated at the end of the experiment.

### Sampling

Rearing tank water samples (40 mL) from each tank for each system, were collected 4, 8, 17, 30 and 46 dph. In addition, one sample from each system was sampled at 1 dph. Incoming water was sampled on the same days except at 46 dph. Live feed samples (rotifers) from the fish tanks were taken at 8 and 17 dph by collecting 100 mL of tank water, rinsing the feed with sterile water in a sterilized sieve and collecting ∼200 rotifers using a sterile syringe. Both water samples and the rinsed live feed samples were filtered through sterile, hollow fiber syringe filters for aqueous solutions (0.2 μm 2.5 cm^2^, DynaGard, Microgon Inc., California) and stored at −20°C. On average 3 cod larvae from each tank were sampled on 8, 17, 30 and 46 dph, by syphoning water through a plastic tube at the middle depth of each tank. Live cod larvae were selected randomly and sacrificed by an overdose of tricaine methanesulfonate (MS222) before further processing. The larvae were rinsed twice in sterilized seawater and transferred individually to Eppendorf tubes, immediately snap frozen in liquid nitrogen and stored at −20°C.

### DNA extraction

DNA was extracted using a DNeasy Blood and Tissue Kit (Qiagen). DNA extraction from individual cod larvae, live feed and water samples was performed as described in the protocol for Gram-positive bacteria by the manufacturer, but with minor modifications (for details, see Attramadal *et al*. 2014).

### PCR amplification and sequencing

Fish, feed and water samples were prepared for Illumina MiSeq sequencing by amplification of the V4 region of the 16S rRNA gene using the following primers (locus-specific V4 primer underlined and bold) including 5′ adapter sequences for later indexing PCR and Illumina MiSeq sequencing:

515 F 5′ TCGTCGGCAGCGTCAGATGTGTATAAGAGACAGNNNN**GTGCCAGCMGCCGCGGTAA** 3′

(Caporaso *et al*. 2011) and

803 R 5′ GTCTCGTGGGCTCGGAGATGTGTATAAGAGACAGNNNN**CTACVVGGGTATCTAAKCCBK**3′. The 803 R primer was designed for this study, because previously published PCR primers targeting this region appear to target and co-amplify algal chloroplast 16S rDNA. Since *Nannochloropsis oculate* algal paste (ReedMariculture) was used in the fish tanks, preliminary PCR and subsequent sequencing for water samples revealed that co-amplification of *Nannochloropsis* 16S rDNA was a major problem. Alignment of *Nannochloropsis oculate* chloroplast and bacterial 16S rRNA gene sequences were used to identify bacteria-specific sequences in the same gene region, and the RDP Probematch tool was used to examine coverage among bacteria.

To obtain approximately the same amount of PCR product for all samples, the reactions were run for 38 cycles for water samples and 40 cycles for cod larval samples (98°C 15 s, 55°C 20 s, 72°C 20 s) with 0.3 µM of each primer, 0.25 mM of each dNTP, 1 mM MgCl_2_, 12 µM of bovine serum albumin (BSA), glycerol (10%), Phusion Hot Start II High-Fidelity DNA Polymerase and reaction buffer from Thermo Scientific in a total volume of 20 µL. PCR products were evaluated on a 1% agarose gel, and purified and normalised using a SequalPrep^TM^ Normalization Plate Kit (Invitrogen). A second PCR was performed to attach dual indices to the normalised amplicons by using the Nextera XT Index Kit. The reactions were run for 8 cycles (98°C 15 s, 50°C 20 s, 72°C 20 s) with 0.25 mM of each dNTP, Phusion Hot Start II High-Fidelity DNA Polymerase and reaction buffer (Thermo Scientific) and 2.5 µl of each index primers in a total volume of 25 µL. The indexed PCR products were purified and normalised as described above, pooled, and concentrated by using Amicon® Ultra-0.5 Centrifugal Filter Devices. The resulting amplicon library was sequenced on two MiSeq lanes (Illumina, San Diego, CA) employing 260 bp paired-end reads at the Norwegian Sequencing Center at the University of Oslo, Norway. The resulting Illumina sequencing data are deposited at the European Nucleotide Archive (accession numbers ERS4778574–ERS4778759).

### DNA sequence data processing

The Illumina sequencing data were processed using USEARCH utility (version 11) (http://drive5.com/usearch/features.html). The command Fastq_mergepairs was used for merging of paired reads, trimming off primer sequences and filtering out reads shorter than 230 base pairs. The processing further included demultiplexing, removal of singleton reads, and quality trimming (the Fastq_filter command with an expected error threshold of 1). Chimera removal and clustering at the 97% similarity level was performed using the UPARSE-OTU algorithm (Edgar 2013). Microbial taxonomy assignment was performed applying the Sintax script (Edgar 2016) with a confidence value threshold of 0.8 and the RDP reference data set (version 16) (Mollerup *et al*. 2016). OTUs (operational taxonomic units) of particular interest were further analysed with the SINA tool at the SILVA web site (www.arb-silva.de). OTUs representing algae, Archaea and *Cyanobacteria/Chloroplast* were removed from the OTU table. An OTU found to represent *Propionibacterium acne*, a well-known contaminant of DNA extraction kits (Mollerup *et al*. 2016) was removed. To remove biases due to variation in sequencing depth, statistical analyses were performed on an OTU table that had been subsampled to 12100 sequencing reads for each sample (the threshold was chosen based on the sample with the lowest number of reads).

### Statistical analysis

Ordination by principal coordinate analysis (PCoA) based on Bray–Curtis similarities (Bray and Curtis 1957) was used to visualise differences in microbial community composition between groups of samples. One-way and two-way PERMANOVA (Anderson 2001) based on Bray–Curtis similarities were used to test for statistically significant differences in microbial community composition between groups of samples. Similarity percentage analysis (SIMPER) was used to identify OTUs responsible for differences (measured as Bray–Curtis similarities) between different groups of samples. Ordination, PERMANOVA (Permutational Analysis of Variance) and SIMPER were performed using the program package PAST version 3.22 (Hammer, Harper and Ryan 2001). Venn diagrams were created using an online tool from Ghent University (http://bioinformatics.psb.ugent.be/webtools/Venn/). Alpha diversity was evaluated as Hill numbers (Tuomisto 2012) with the *reyni* function from the *vegan* package in R (https://cran.r-project.org/web/packages/vegan/index.html, version 2.5–6). Richness, or diversity of order 0, counts the number of OTUs in each sample, while evenness was defined as diversity of order 1 divided by diversity of order 0. MEGA X software (Kumar *et al*. 2018) was used to align OTU sequences by the Muscle algorithm and to make a neighbour-joining phylogenetic tree of the OTUs in the dataset.

### Estimation of significance of ecological processes

To evaluate the bacterial community assembly in individual fish and water samples, the nearest taxon index (NTI) was calculated, as described by Webb (2000). NTI values >2 indicate that the community is more phylogenetically clustered than expected by chance, and thus that deterministic processes such as environmental selection have structured the community (Zhou and Ning 2017). NTI was calculated for each water and fish sample with the function ses.mntd(null.model = ‘taxa.labels’, abundance.weighted = FALSE) in the R-package picante (version 1.8) (Kembel *et al*. 2010). To investigate the ecological processes causing inter-individual variation in the cod larval communities, we used a null-model based statistical framework based on β-NTI, which reflects the phylogenetic dissimilarity between two microbial communities (Stegen *et al*. 2015). β-NTI was calculated within each system and sampling day. With β-NTI values <−2, the communities have similar phylogenetic clustering patterns, and it is assumed that community assembly is caused by homogenous selection. β-NTI values >2 indicate that the communities are under heterogenous selection pressure. For β-NTI values not significantly different from the null model (i.e. |β-NTI| < 2), the community assembly is primarily assumed to be driven by stochastic processes, such as drift, homogenising dispersal and dispersal limitation acting with drift.

## RESULTS

Atlantic cod larvae were reared with three different microbial metacommunities resulting from three independently operated water treatment systems: a traditional FTS, an MMS and an RAS. After quality trimming and chimera removal, we obtained 2 378 168 sequence reads from 16S rRNA amplicon sequencing of water, feed and fish samples. A total of 3371 OTUs were detected after subsampling to an equal number of amplicon reads (12 100) for each sample.

### Alpha diversity

Comparing the estimated number of OTUs (Chao1; Supplementary Table S2, see online supplementary material), with the observed OTU richness (Supplementary Fig. S1, see online supplementary material) revealed that the sequencing depth covered approximately 85, 67 and 75% of the estimated total richness for fish, tank water and feed samples, respectively. The observed OTU richness in tank water typically exceeded that in young larvae (8 dph) by a factor 2.5, while at 46 dph it was only 1.8 times higher. The richness was more stable for tank water bacteria than for fish bacteria in all rearing systems throughout the experiment. Interestingly, for fish bacteria both the observed richness and the evenness (Supplementary Fig. S1) was lowest at the two earliest sampling points and increased with age. This was particularly pronounced for the evenness, which increased more than 10 times from 8 to 46 dph in FTS. In RAS, the evenness was higher at 8 dph than at 17 and 30 dph.

### Environmental bacterial communities

The source bacterial community included the bacterial communities in the incoming water, in the tank water, associated with the feed and associated with the fish at the time of transfer to the rearing tanks. All systems received water from the same source and feed from the same cultivation tank. Thus, all OTUs detected can be considered a global species pool. Ecological processes within the three systems further structured the three metacommunities.

#### The incoming bacteria and selection structured the tank water communities

**Figure 1.**
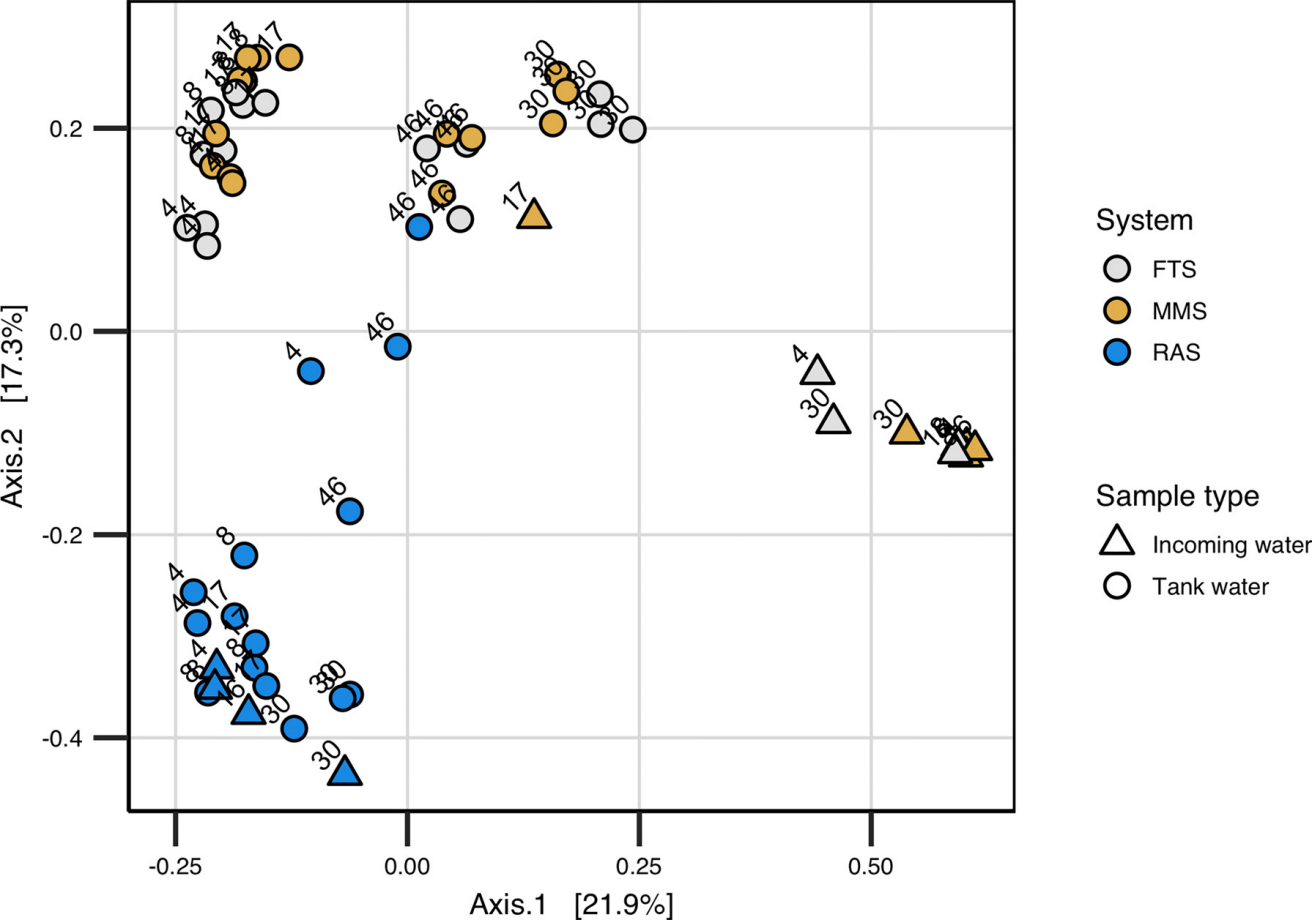
PCoA ordination plot based on Bray–Curtis similarities for comparison of the bacterial communities of tank water and incoming water at 4, 8, 17, 30 and 46 dph (day 4–day 46) in FTS, MMS and RAS. All tanks received MMS water from 31 dph onwards. Colours indicate the water treatment system, numbers in plot indicate the sampling-day and shapes the sample type (triangle = incoming water, circle = tank water).

The different water treatment systems yielded significantly different bacterial communities in the water going into the fish tanks (Bray–Curtis similarity indices, one-way PERMANOVA, *P* ≤ 0.03). The bacteria of the incoming water clearly affected the composition of the tank water communities in all systems (Fig. 1). The communities in the incoming water and the tank water was similar in RAS (Fig. 1), whereas for both MMS and FTS the communities of the incoming water differed significantly from those of the tank water (Bray–Curtis similarity indices, one-way PERMANOVA, *P* < 0.002). This indicates that the microbes in the water were under different selection pressures in the FTS and MMS tanks compared to the incoming water in these systems. The tank water communities in RAS differed significantly from those in FTS and MMS (Bray–Curtis similarity indices, one-way PERMANOVA, *P* = 0.0001). However, there were no significant differences in tank water communities between FTS and MMS ( *P* = 0.7). At 46 dph, when all systems had received identical incoming water (MMS) for 2 weeks, the bacterial communities of the tank water appeared to be more similar between RAS and FTS/MMS than earlier in the experiment (Fig. 1). This was corroborated by average Bray–Curtis similarities (Supplementary Fig. S2, see online supplementary material), with significantly higher similarity between RAS and FTS tank water, and RAS and MMS tank water at 46 dph than at 30 dph (t-test, *P* = 0.002 and 0.0004, respectively). This indicates that both the composition of the bacterial communities in the incoming water and the selection in the fish tanks had an impact on the composition of the tank water communities. Moreover, the water communities in each replicate tank was more phylogenetically structured than expected by stochastic assembly (NTI > 2), indicating that they were assembled by deterministic processes such as selection (Supplementary Fig. S3, see online supplementary material).

At the OTU level, the bacterial community composition of the tank water varied considerably over time and between systems (Supplementary Fig. S4, see online supplementary material). As an example, OTU_6 (classified as *Leucothrix* by the SILVA database classification tool) dominated in the FTS and MMS tank water at 8 dph, accounting for around 30% of the reads. Its relative abundance decreased dramatically at 30 dph. In RAS, OTU_6 was low in abundance throughout the experiment (∼0.25% of the reads). Other examples are a high abundance of an *Aliivibrio* OTU (OTU_28), exclusively in the FTS tank water at 30 dph (average 21% of the reads), and a predominance of a *Polaribacter* OTU (OTU_14) in RAS tank water, with maximum abundance at 30 dph (∼40% in two of the replicate tanks).

#### The bacterial comminities associated with the feed were influenced by the tank water communities

**Figure 2.**
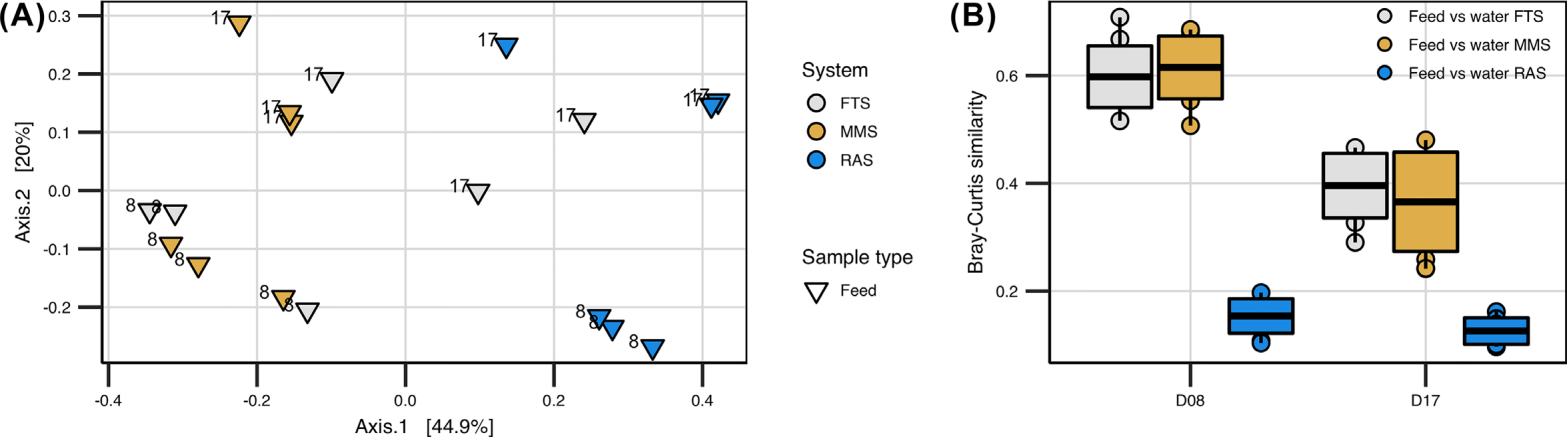
Comparison of the composition of the bacterial communities in feed and water samples from the tanks for the different water treatment systems (FTS, MMS and RAS) taken at 8 and 17 dph. **(A)** PCoA ordination plot based on Bray–Curtis similarities. Colours indicate the water treatment system and numbers in plot the sampling-day. **(B)** Bray–Curtis similarities of the bacterial communities between feed and water within each system at each sampling-time. Each box is based on nine comparisons (three water and three feed samples). Solid black line indicates mean similarity and the surrounding box the standard deviation.

All live feed distributed to the fish tanks originated from the same cultivation tank. Thus, we assumed that the bacterial communities of the feed introduced to the systems were identical. However, rotifers actively ingest bacteria, and the microbial communities may change before the feed is eaten by the fish. The bacterial community of the feed sampled from the RAS rearing tanks differed significantly from those in FTS and MMS rearing tanks (one-way PERMANOVA; *P* = 0.015 and 0.0033 for comparison with FTS and MMS, respectively). There were no significant differences in the bacterial communities of the feed between the FTS and the MMS (*P* = 0.8, Fig. 2A), which indicates that the tank water communities influenced the feed communities. However, the tank water and feed communities differed significantly in each of the systems at each sampling time (one-way PERMANOVA, *P* < 0.02), indicating that either selection or dispersal were dominating the community assembly of the live feed. The Bray–Curtis similarity between the feed and water samples was approximately three times lower in RAS compared with FTS and MMS (Fig. 2A and B).

### Selection in the host contributed to the bacterial community assembly of the fish

**Figure 3.**
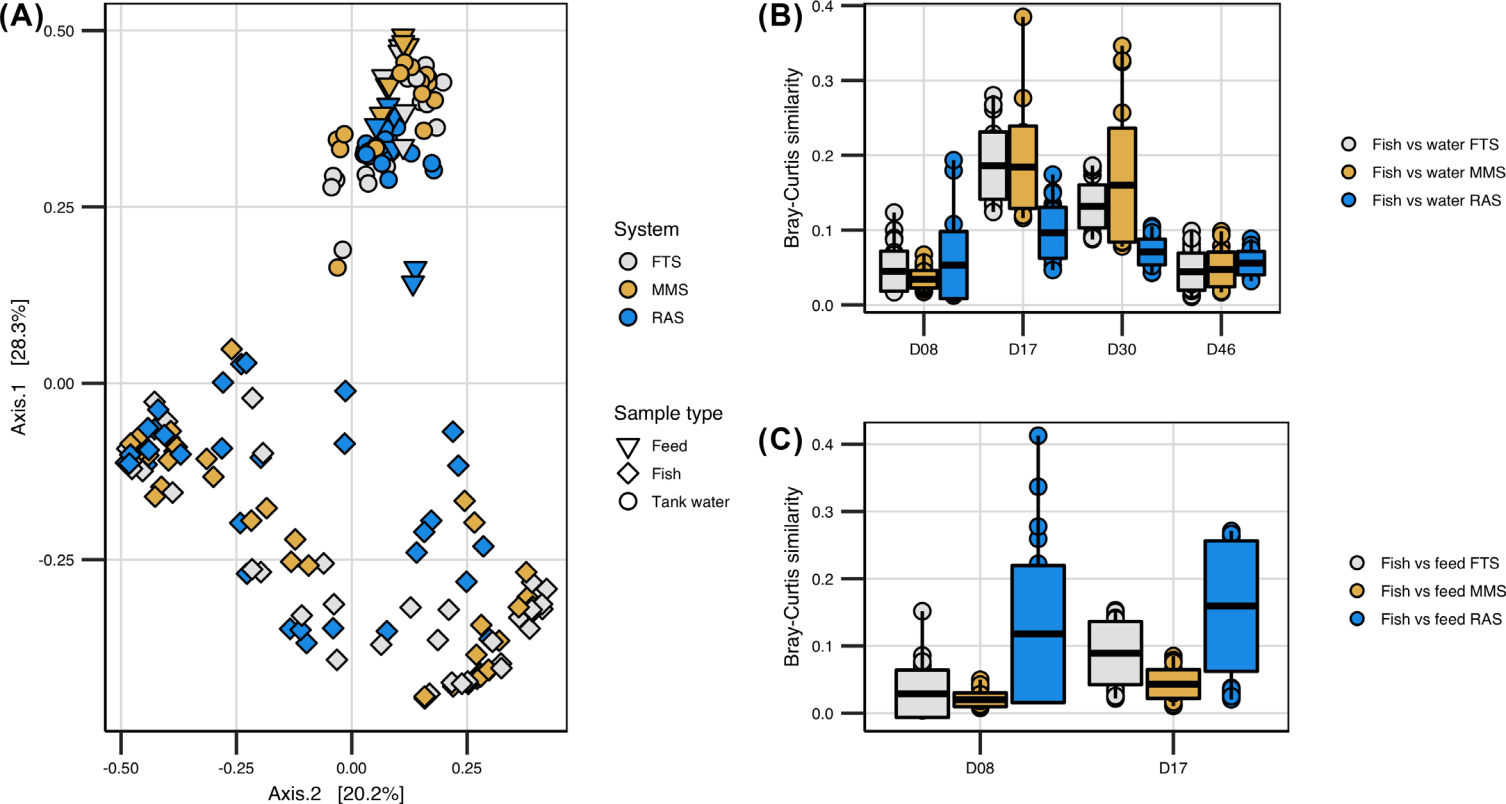
Comparison of bacterial communities of fish, water and live feed samples. **(A)** PCoA ordination plot based on Bray–Curtis similarities for comparison of bacterial communities of all fish, water and feed samples in all treatment systems throughout the experiment. Colours indicate the water treatment system and shapes the sample type (triangle = feed, diamond = fish larvae and circle = tank water). **(B)** Bray–Curtis similarities for comparison of fish and tank water bacterial communities in FTS, MMS and RAS at 8, 17, 30 and 46 dph. Each box is based on 27 comparisons (three water and nine fish samples). Solid black line indicates mean similarity and the surrounding box the standard deviation. **(C)** Bray–Curtis similarities for comparison of communities of fish and feed samples in FTS, MMS and RAS at 8 and 17 dph. Each box is based on 27 comparisons (three feed and nine fish samples). Solid black line indicates mean similarity and the surrounding box the standard deviation.

**Figure 4.**
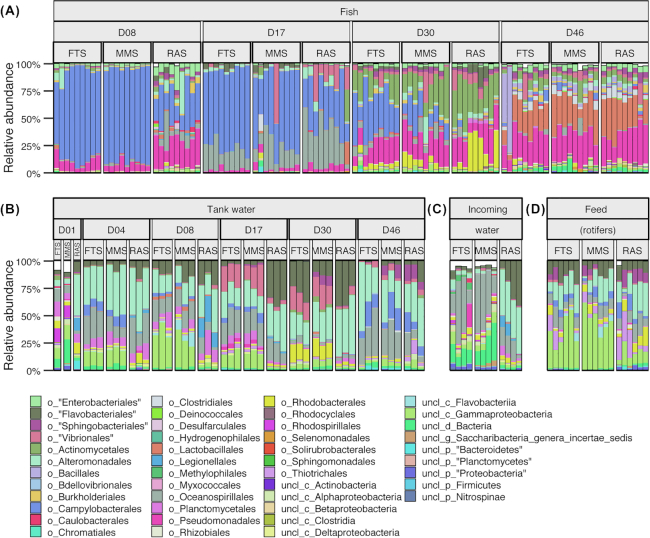
Relative abundances of the 45 most abundant bacterial orders detected in **(A)** fish, **(B)** tank water, **(C)** incoming water and **(D)** feed samples in the systems FTS, MMS and RAS; D01-D46 indicates sampling times given as dph.

The bacterial communities of the fish were highly dissimilar from those of the tank water and live feed throughout the experiment, as reflected by both PCoA ordination (Fig. 3A) and the community composition at the order level (Fig. 4). Average Bray–Curtis similarities for water/feed vs fish comparisons within systems and sampling times ranged from 0.0013 to 0.22 (including standard deviations in Fig. 3B and C). The differences were significant for all systems at all sampling times (one-way PERMANOVA, *P* < 0.006 and p ≤ 0.005 for water and feed comparisons, respectively). Interestingly, as much as 63% of all OTUs observed in the fish were unique for fish samples (Supplementary Fig. S5, see online supplementary material). Moreover, NTI values for individual fish communities indicated more phylogenetic clustering than expected for stochastic community assembly (Supplementary Fig. S3). This implies that selection was important for community assembly within the fish.

### Stochastic processes contributed to variation in the bacterial communities between individual cod larvae

**Figure 5.**
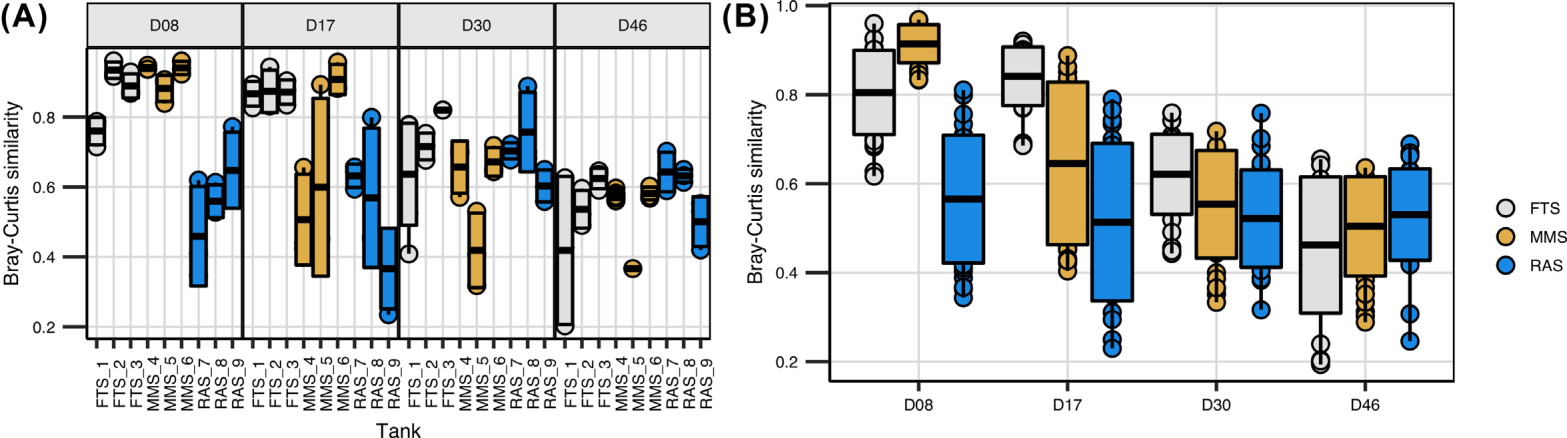
Bray–Curtis similarities for comparisons of bacterial communities of individual fish within **(A)** and between **(B)** replicate rearing tanks (FTS1–3, MMS4–6 and RAS7–9) at 8, 17, 30 and 46 dph (D8–D46). Comparisons are based on between two and four individuals from each tank (A) or nine samples from each system and sampling time (B). For FTS at 30 dph and MMS at 46 dph, only two cod larvae were sampled from one of the tanks, but the total number of fish sampled from each system was always nine. Solid black line indicates mean similarity and the surrounding box the standard deviation.

**Figure 6.**
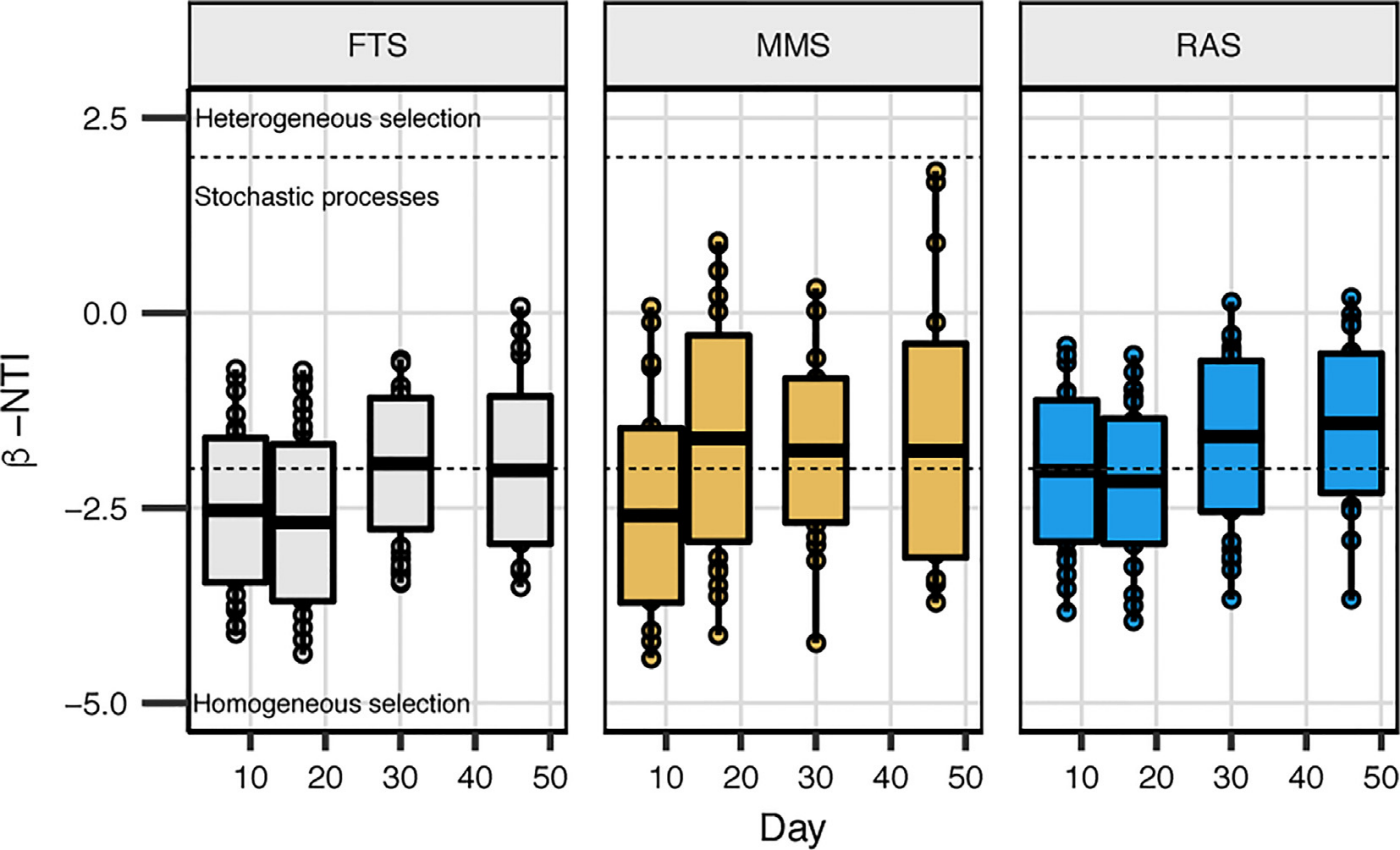
β-NTI for comparisons of fish bacterial communities between individuals within each system (FTS, MMS and RAS) at each sampling time. β-NTI > 2 and < −2 indicates heterogeneous and homogeneous selection, respectively. |β-NTI| < 2 represents comparisons that are not significantly different from the null model and indicate stochastic community assembly. Solid black lines indicate the mean β-NTI value on a sampling day within a treatment system (*n* = 36), and the surrounding box the standard deviation.

Average Bray–Curtis similarities show that the fish bacterial communities varied among individuals in the same rearing tank and between replicate tanks (i.e. within treatments), and especially at 30 and 46 dph (Fig. 5A and B, respectively). This indicates that processes such as drift or heterogenous selection in the individual larvae and rearing tanks also contributed to the community assembly in the fish. The β-NTI analysis indicated that there was a temporal increase in the contribution of stochastic processes to the difference in community structure between individual fish (Fig. 6 and Supplementary Fig. S6, see online supplementary material). This increase suggests that selection within the hosts was most important during the early rearing life stages, and that stochasticity created variation in the communities between fish in the same system and with increasing importance over time. This temporal trend was most pronounced in RAS, with a gradual increase in the relative contribution of stochastic processes from 41 to 75%. None of the comparisons were categorised as heterogeneous selection (β-NTI > 2), indicating that the selection pressure was similar among individual hosts, and that the inter-individual variation was due to stochastic processes.

### The bacterial communities of the water affected the bacterial communities of the fish

**Figure 7.**
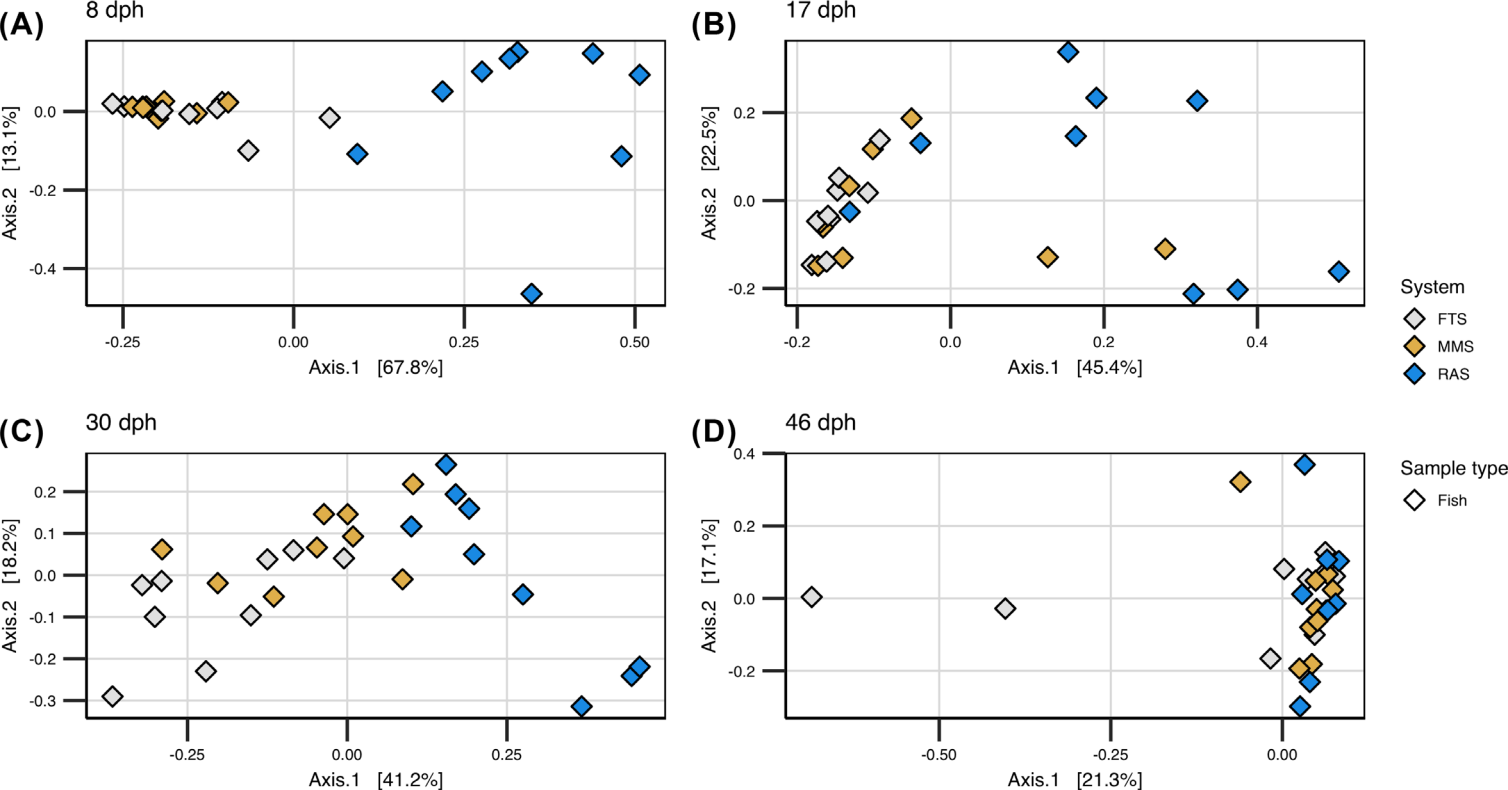
PCoA ordination plot based on Bray–Curtis similarities for comparison of fish bacterial communities in FTS, MMS and RAS at **(A)** 8, **(B)** 17, **(C)** 30 and **(D)** 46 dph. Colours indicate water treatment system.

Despite the high dissimilarity between bacterial communities in tank water and fish, the fish communities differed according to rearing system. This indicates that the bacteria in the water influence the bacterial communities of the fish. The fish communities in RAS differed significantly from those in FTS and MMS at 8, 17 and 30 dph (one-way PERMANOVA *P* < 0.03) (Fig. 7A–C), with average Bray–Curtis similarities of around 0.4–0.5 for between-system comparisons (Supplementary Fig. S7, see online supplementary material). For FTS and MMS, however, the fish communities were more similar (average Bray–Curtis similarities of 0.6–0.9, Supplementary Fig. S7), and differed significantly only at 30 dph (one-way PERMANOVA, *P* = 0.02). Interestingly, at 46 dph, when all fish tanks had received the same incoming water (MMS) for 16 days, there were no significant differences in the fish communities between any of the systems (Fig. 7D). Moreover, the average Bray–Curtis similarities were comparable within and between systems (Fig. 5 and Supplementary Fig. S7). This clearly points to an influence of the environmental bacteria on the fish communities. Next, we investigated the metacommunities in more detail to elucidate the influence of the water bacteria on the fish communities at the OTU level. Correlating the number of reads for each OTU in both fish and tank water samples (Supplementary Fig. S8a-c, see online supplementary material), revealed that only five OTUs in the whole data set reached average abundances larger than 2% (of the total reads in at least one sample) for both fish and water samples in at least one system and sampling time (Supplementary Table S3, see online supplementary material). This implies that distinct selection regimes act on the water and the fish bacteria, and that few bacterial populations were selected for in both environments. Only OTU_3 ( *Marinomonas*) and OTU_13 (*Aliivibrio*) were more abundant than 5% in both sample types (Supplementary Table S3). The *Marinomonas* OTU was abundant in both water and fish samples at 17 dph in all systems. A SIMPER analysis identified the OTUs contributing most to the differences in fish communities between RAS and FTS/MMS and we identified the average abundance of these OTUs in the relevant water samples (Supplementary Table S4, see online supplementary material). An OTU representing *Arcobacter* (OTU_1) contributed most to the dissimilarity at both 8, 17 and 30 dph (explaining 46, 33 and 21% of the Bray–Curtis dissimilarity, respectively). This OTU dominated the fish samples in MMS and FTS at 8 and 17 dph (average 81 and 65% of the total reads, respectively), and was almost 20 times more abundant in FTS/MMS larvae than in RAS larvae at 30 dph. It was also far more abundant in the water in FTS and MMS compared with RAS at 8 and 17 dph (∼40 times), but the maximum abundance never exceeded 2.3% on average in the water of any system on these days. Thus, even though highly distinct bacterial communities were selected for in water and fish, differences in the relative abundance of rare water OTUs seemed to have a major impact on the bacterial communities of the fish. This supports the above-mentioned conclusion regarding the significance of selection for bacteria associated with the fish.

## DISCUSSION

In this study, we used cod larvae as the model organism and investigated the bacterial community assembly during a first feeding experiment. The fish were reared with three different metacommunities (FTS, MMS and RAS) for 30 days, followed by a period of 16 days where all tanks received the same microbiota (MMS). We aimed at quantifying the relative importance of the four high-level ecological processes described by Vellend (2016) and at elucidating the relationship between the bacteria in water and associated with the fish.

### Different selection regimes resulted in different bacterial communities in the water

The water treatments differed between the three systems and yielded three incoming waters with distinct bacterial communities. However, the communities in FTS and MMS tank water were not significantly different, but there was variation at the OTU level (Supplementary Fig. S4). In these systems the carrying capacity of the incoming water was higher than in the fish tanks and this promotes fast-growing opportunistic bacteria (Attramadal *et al*. 2014; Vadstein *et al*. 2018). This opportunistic selection most likely caused the similar community composition in the two systems (Vadstein *et al*. 2018). However, at 32 dph the survival of cod in MMS was ∼65% higher than in FTS (results calculated by and presented in Attramadal *et al*., 2014). Except for the metacommunity composition, the FTS and MMS fish were reared under equal conditions. Therefore, detrimental fish–microbe interactions are the most likely explanation for the difference in survival. In RAS systems, on the other hand, the carrying capacity is similar throughout the system. This restricts opportunistic growth in the fish tanks, and consequently the microbiota in the incoming water and the tank water are similar, as shown by Attramadal *et al*. (2014). Consumption of dissolved organic matter mainly in the biofilters under strong competition, long hydraulic retention time and absence of disinfection created K-selection in RAS water (MacArthur and Wilson 1967; Attramadal *et al*. 2014; Vadstein *et al*. 2018). Thus, both the composition of the incoming water microbiota and the selection forces in the fish tanks contributed to the bacterial community assembly in the tank water.

### Bacterial community assembly in cod larvae was dominated by selection and drift

We showed that the bacterial communities of the fish were highly dissimilar from the bacterial communities of the water and feed, indicating that selection was important for community assembly in the fish (Yan, van der Gast and Yu 2012; Bakke *et al*. 2015; Yan *et al*. 2016). This was validated as all NTI values indicated strong phylogenetic clustering and that 63% of the OTUs were unique to the fish. Because all β-NTI values were <2 we ruled out heterogeneous selection as the dominating process steering the bacterial community assembly of the fish. While many analyses indicated strong selection, we observed considerable inter-individual variations both within tanks and between tanks in the same system and the variations seemed to increase with larval age (Figs. 5 and 6). While homogeneous selection should reduce variation, stochastic processes such as dispersal, diversification and drift introduce a randomness that increases variation. Based on traditional metacommunity theory, microbes disperse from the water to the fish and from the fish to the water through excretion (Miller, Svanbäck and Bohannan 2018). Through water, the microbes can disperse from one fish to another given that the fish are in the same tank. In this experiment the major source pools of bacteria to the systems were the incoming water and the feed. The tank water and feed microbiota are the two primary sources of bacteria for fish. Marine fish larvae actively take up bacteria from the surrounding water at rates 100 times higher than the drinking rate, resulting in a consumption of 10^4^–10^6^ bacteria per larva per day (Reitan, Natvik and Vadstein 1998; Vadstein *et al*. 2018). Ingestion of feed provides an additional 10^5^–10^7^ (Reitan, Natvik and Vadstein 1998). Consequently, it is unlikely that there was dispersal limitation from the environment to the larvae. Given the length of the experiment we believe it is unlikely that diversification played a role in community assembly at the OTU level (Burns *et al*. 2016). We therefore argue that the processes we have classified as stochastic are equal to drift. β-NTI calculations showed that there was no heterogeneous selection, thus indicating that drift was the main driver of the inter-individual variation observed between larvae. Based on our findings it appears that selection had a major role in structuring the metacommunity, while drift created variation within it. On average, selection within the fish and drift contributed equally to the bacterial community assembly in the cod larvae (Supplementary Fig. S6).

Investigations on community assembly in fish larvae have been done previously (Yan, van der Gast and Yu 2012; Burns *et al*. 2016; Yan *et al*. 2016) and the methods used are either based on composition (neutral model) or phylogeny (β-NTI null-model). In a study on zebrafish, Burns *et al*. (2016) based their estimations on composition, and they argue, as we do, that stochastic processes generate considerable inter-individual variation. However, their results showed that the contribution of stochasticity decreased with host age. In other studies (Yan, van der Gast and Yu 2012; Yan *et al*. 2016) where phylogenetic-based models have been used, as has been done in our study, the results have shown that the contribution of stochasticity increases with host age. Different microbial species in communities may result in large differences between communities when using the neutral model, however if the comparison is based on phylogenetic models the differences might be smaller if the phylogenetic distance between the species is short. This might contribute to explaining the different conclusions drawn by Burns and co-workers and by us. While Burns *et al*. (2016) included the whole life cycle of the zebrafish in their study, we only examined the larval life stage of Atlantic cod. The seemingly contradictory results might also reflect biological significant differences.

### Rare OTUs in the bacterial communities of the water may have large consequences for the community assembly of the fish

We have previously shown that it is possible to promote mutualistic fish–microbe interactions in aquaculture systems through well-designed water treatment based on ecological principles (Vadstein *et al*. 2018). In concordance with our previous study (Vestrum *et al*. 2018), we demonstrated here that the bacterial communities of the tank water significantly affected the communities of the fish. From 31 dph onwards, all fish were exposed to the same species pool, and we observed that the differences in the bacterial communties of the fish were reduced between systems. This indicates that the water microbiota affected the bacterial communities of the fish even after the initial colonisation had resulted in different bacterial communities of the fish in the different systems. However, at this point the fish microbiota had probably not yet reached maturity. Therefore, we cannot rule out that other factors such as the developmental stage and the change of feed at 31 dph affected the succession of the bacterial communities as well. The immaturity of the microbiota might also explain the evenness and richness increasing with age. The influence of the water microbes on the bacterial communities of the fish seems to be in contrast with the large dissimilarity observed between the bacterial communities of the fish and the water. The experimental design in this study, including three different microbial water qualities with triplicate tanks, and detailed characterisation of bacterial communities in both fish and water, allowed us to investigate this paradox. Most OTUs found in the fish had low abundances in the water, and vice versa, indicating distinct selection regimes for these two environments. However, a few OTUs were present in relatively high abundances in both water and fish, suggesting that minor fractions of the bacteria were able to compete in both environments. Moreover, we found that an OTU representing *Arcobacter* (a potential opportunistic pathogen (Fitzgerald and Nachamkin 2015) was responsible for most of the differences observed between the bacterial communities of fish in RAS and FTS/MMS. This OTU constituted as much as 81 and 65% of the total reads for FTS and MMS fish samples, respectively, at 8 and 17 dph. The survival of cod larvae was 40% lower in the FTS than in MMS and RAS. This, as well as the excessive dominance of the *Arcobacter* OTU in FTS and MMS larvae, might be explained by differences in the structure of the environmental bacterial communities between the systems, with higher fractions of opportunists in FTS/MMS, and the resulting implications for microbe–microbe and microbe–host interactions. As proposed by Miller *et al*. (2018), the host health might have been affected by the microbiota. In our study, the fish reared in FTS and MMS may have become more susceptible to colonisation by *Arcobacter* due to detrimental host–microbe interactions resulting from the presumably higher fraction of opportunistic bacteria in the rearing water in these systems than in RAS. This shows that the systems’ metacommunity should be considered when investigating the community assembly in hosts. Moreover, this study suggests that it is possible to steer the metacommunity towards mutualistic host–microbe interactions.

Sequencing of 16S rDNA amplicons and data processing involving OTU clustering has been the golden standard for microbial diversity studies. However, new approaches have been developed in recent years. For example, as an alternative to the OTU clustering of similar 16S sequences, the concept of amplicon sequencing variants (ASV) has been introduced (Porter and Hajibabaei 2018). ASV-based studies have been suggested to give more realistic and detailed characterisations of microbial communities compared to OTU-based studies (Porter and Hajibabaei 2018). It would be interesting to investigate how this would influence studies on ecological processes in microbial ecology, with the potential for reflecting the actual microbial diversity to a greater extent.

Through a detailed characterisation of the bacterial communities of cod larvae and their environment we were able to elucidate the relationship between host and environmental bacteria. In aquaculture, cod larvae live in a microbial metacommunity that receives bacteria from incoming water and feed. This metacommunity was strongly structured by selective forces, but drift created variation. We were able to identify a single OTU that was selected for in both FTS and MMS and was highly abundant in the fish microbiota in these systems. This OTU might have influenced the survival of the larvae. These findings suggest that it is possible to steer the metacommunity towards mutualistic host–microbe interactions.

## Supplementary Material

fiaa163_Supplemental_File
