# Supplementary material for: Bacterial community assembly in Atlantic cod larvae (Gadus morhua): contributions of ecological processes and metacommunity structure
Source: FEMS Microbiol Ecol. 2020 Aug 20;96(9):fiaa163. doi: 10.1093/femsec/fiaa163 (PMC7456331; doi:10.1093/femsec/fiaa163)
Supplement: fiaa163_Supplemental_File [file fiaa163_supplemental_file.docx]

# Supplementary material

# Bacterial community assembly in Atlantic cod larvae (*Gadus morhua*): Contributions of ecological processes and metacommunity structure.

Ragnhild I. Vestrum^a^, Kari J.K. Attramadal^a^, Olav Vadstein^a^, Madeleine Stenshorne Gundersen^a^ and Ingrid Bakke^a#^.

^a^Department of Biotechnology and Food Science, NTNU - Norwegian University of Science and Technology, Trondheim, Norway

Running title: Bacterial community assembly in cod larvae.

#Address correspondence to Ingrid Bakke, [Ingrid.bakke@ntnu.no](mailto:Ingrid.bakke@ntnu.no)

Table S1: Overview of the rearing conditions from 0-46 days post hatching. The larvae were kept in darkness until mouth opening 3 days post hatching (dph), and in continuous light thereafter. From 2 – 24 dph *Nannochloropsis oculata* algae paste (Reed Mariculture) was added to the rearing tanks (1 mg C L^-1^ final concentration). The cod larvae were fed rotifers (*Brachionus* *plicatilis*, Cayman) from day 3 to 26 dph, and from 22 – 32 dph the fish were fed *Artemia* nauplii, hatched from INVE EG cysts. The live feed was distributed in the tanks by a robot system (Storvik Aqua AS) 4 – 6 times per day, to tank densities of 5000 –12,000 rotifers L^−1^ and 3000-5000 *Artemia* L^-1^, respectively. From day 31 the fish were fed formulated feed (GEMMA Micro, SKRETTING, Norway) by the same robot system, 4 times per hour at a gradually increasing level of 3 – 15 g tank^-1^ per day.

| **Days** | 0 | 1 | 2 | 3 | 4 | 5 | 6 | 7 | 8 | 9 | 10 | 11 | 12-21 | 22-24 | 25-26 | 27-30 | 31-32 | 33-39 | 40-46 |
| --- | --- | --- | --- | --- | --- | --- | --- | --- | --- | --- | --- | --- | --- | --- | --- | --- | --- | --- | --- |
| **Temp (°C)** | 6 | | 7 | | 8 | | 9 | | 10 | | 11 | | 12 | | | | | | |
| **Light** | Off | | | On | | | | | | | | | | | | | | | |
| **Feed** |  | | Algae paste | | | | | | | | | | | |  | | | | |
|  |  | | | Rotifers | | | | | | | | | | | |  | | | |
|  |  | | | | | | | | | | | | | *Artemia* nauplii | | | |  | |
|  |  | | | | | | | | | | | | | | | | GEMMA Micro | | |
|  | **Water treatments** | | | | | | | | | | | | | | | | | | |
| **FTS** | UV filtered water | | | | | | | | | | | | | | | | Microbially  matured water | | |
| **MMS** | UV + Microbially matured water | | | | | | | | | | | | | | | |  |  |  |
| **RAS** | Recirculation water | | | | | | | | | | | | | | | |  |  |  |


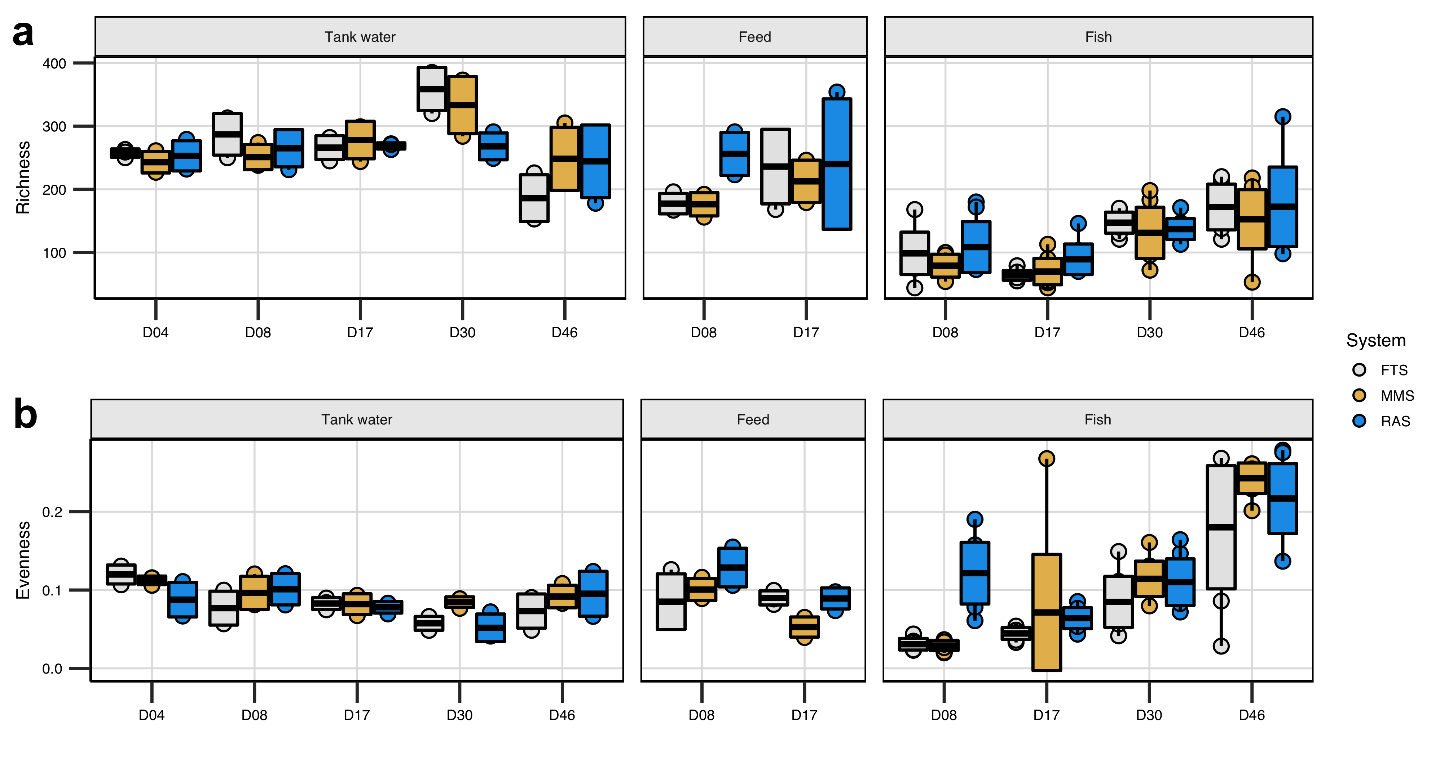


Figure S1: a) Observed OTU richness and b) evenness for bacteria in tank water, feed and larvae samples. Average values are based on 3 samples for water and feed, and 9 for fish. D4 – D46 stands for 4 – 46 dph. Error bars are standard deviations.

Table S2: Average observed richness and average Chao1 values for fish, water and feed samples, in FTS, MMS and RAS.

|  | **Fish** | | |
| --- | --- | --- | --- |
|  | **FTS** | **MMS** | **RAS** |
| Observed richness | 120.6 | 108.3 | 126.9 |
| Chao1 | 145.5 | 126.1 | 144.2 |
|  | | | |
|  | **Water** | | |
|  | **FTS** | **MMS** | **RAS** |
| Observed richness | 294.5 | 312.2 | 267.8 |
| Chao1 | 458.5 | 452.3 | 392.4 |
|  | | | |
|  | **Feed** | | |
|  | **FTS** | **MMS** | **RAS** |
| Observed richness | 195.1 | 182.9 | 225.0 |
| Chao1 | 273.8 | 241.5 | 287.6 |


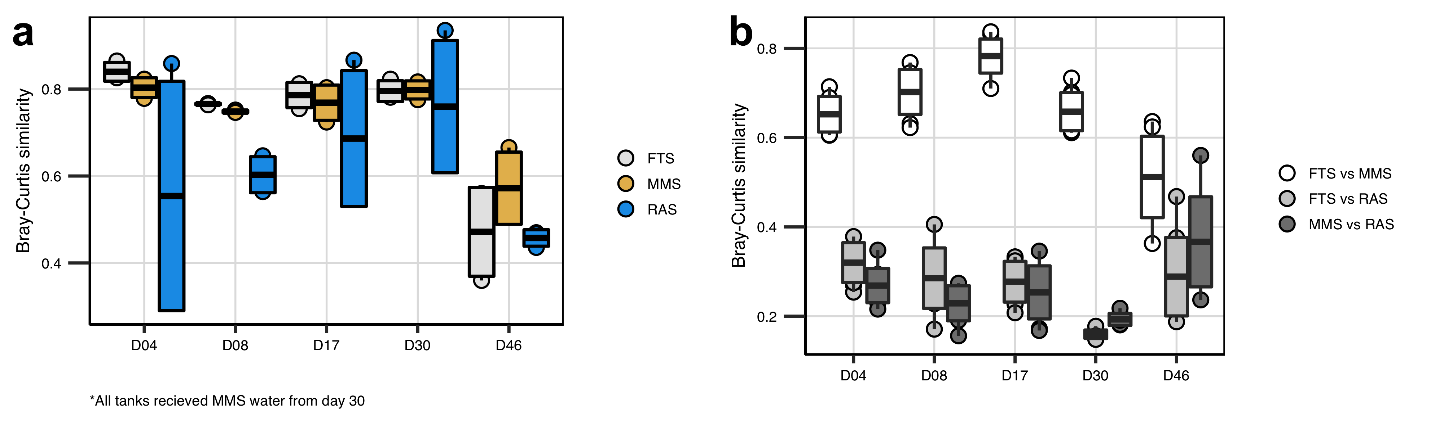


Figure S2: Average Bray-Curtis similarities for comparisons of the bacterial community composition of the tank water at 4, 8, 17, 30 and 46 dph (Day 4 – Day 46) a) between replicate tanks within water treatment systems (FTS, MMS and RAS) and b) between water treatment systems. Average values are based on 3 water samples from each system on each sampling day. Error bars are standard deviations.


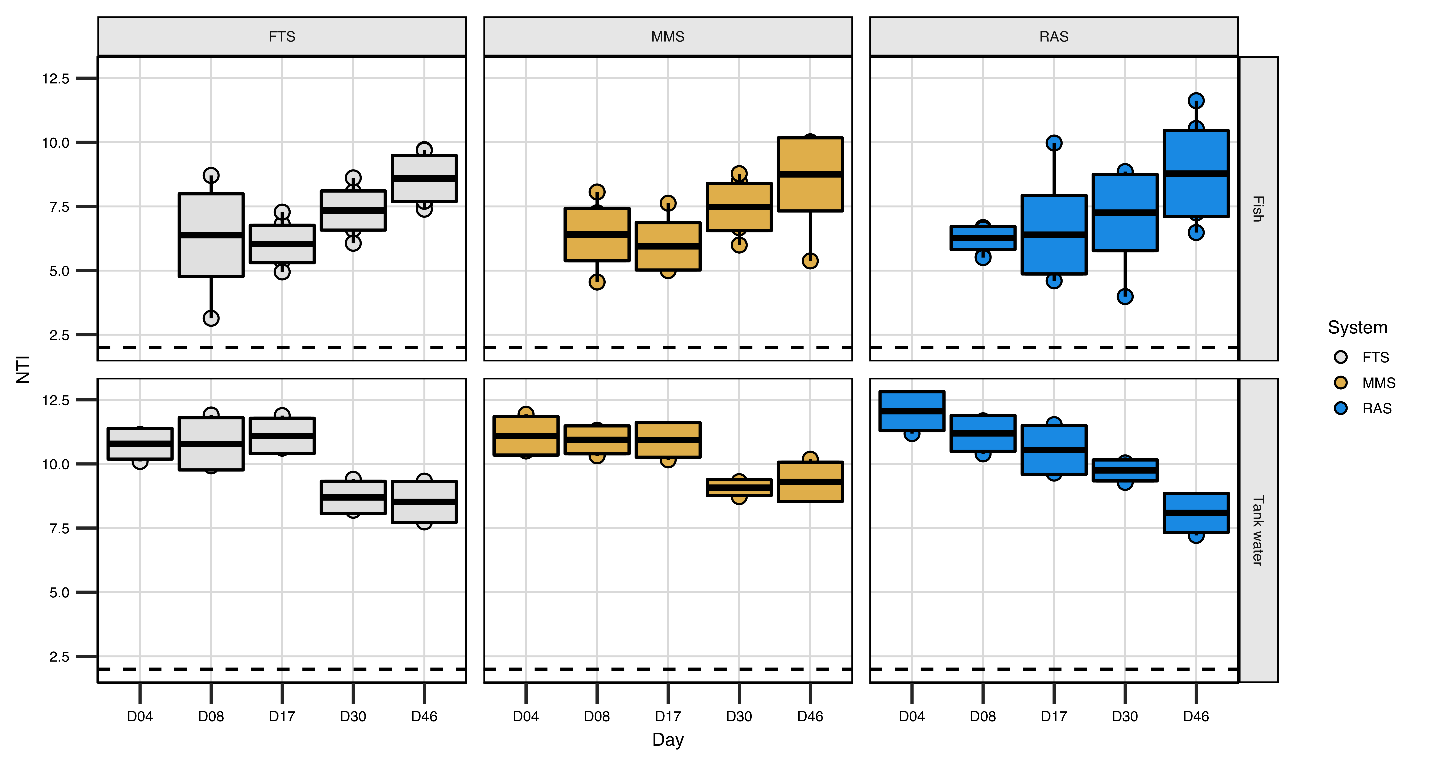


Figure S3: Nearest taxon indec (NTI) value for each local community in fish and water samples. NTI values > 2 indicate phylogenetic clustering, in which OTUs are more closely related than by chance. Negative NTI values indicate phylogenetic overdispersion. |NTI| < 2 reflect that local phylogenetic composition is not significantly different from the null model distribution. Solid black lines indicate the mean NTI value at a sampling day within a sample type (n = 9 for fish and n = 3 for water), and the surrounding box the standard deviation.


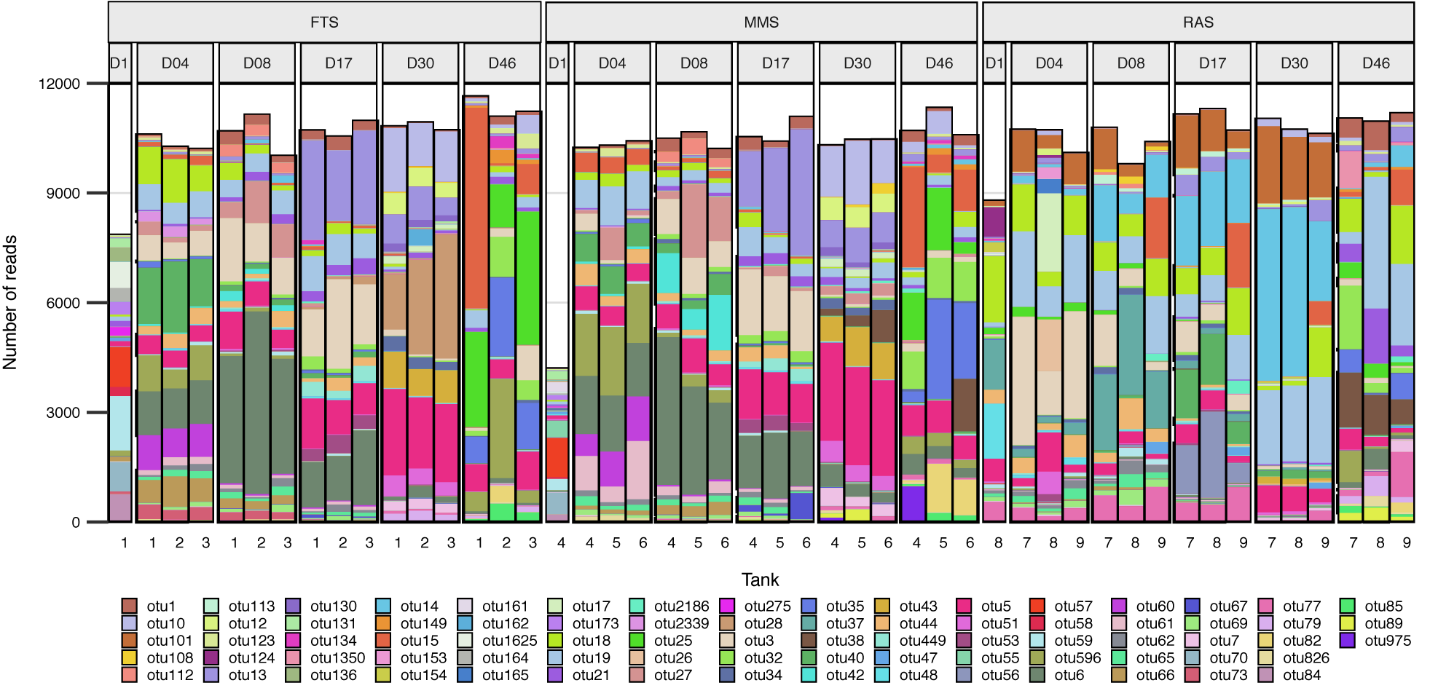


Figure S4: Number of reads of OTUs detected in the tank water (>2% in at least one sample) in three replicate tanks of FTS (1-3), MMS (4-6) and RAS (7-9) at 1-46 dph (D1-D46).


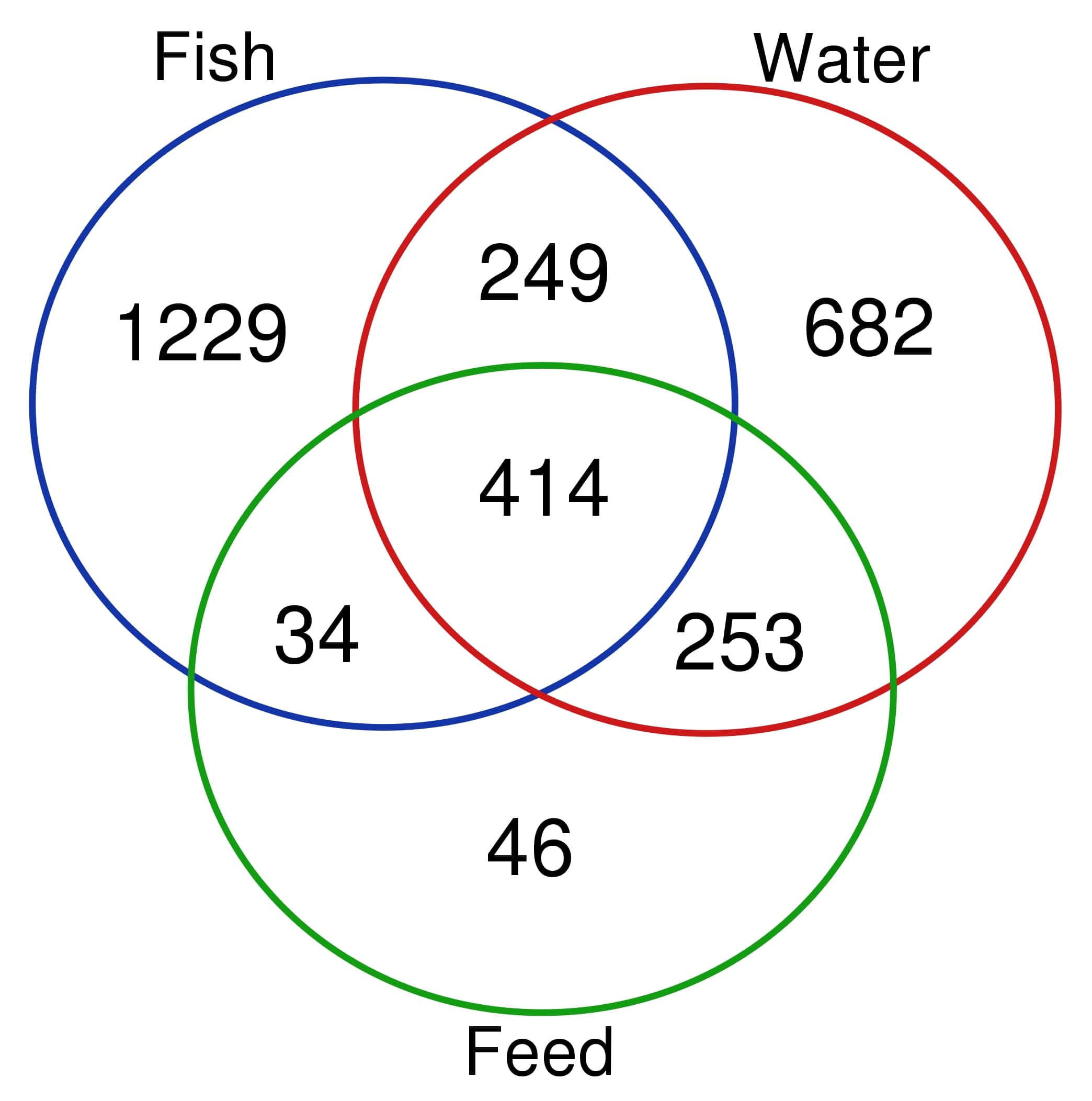


Figure S5: Venn diagram showing the number of detected OTUs unique for and shared by different sample types (fish, water and feed).


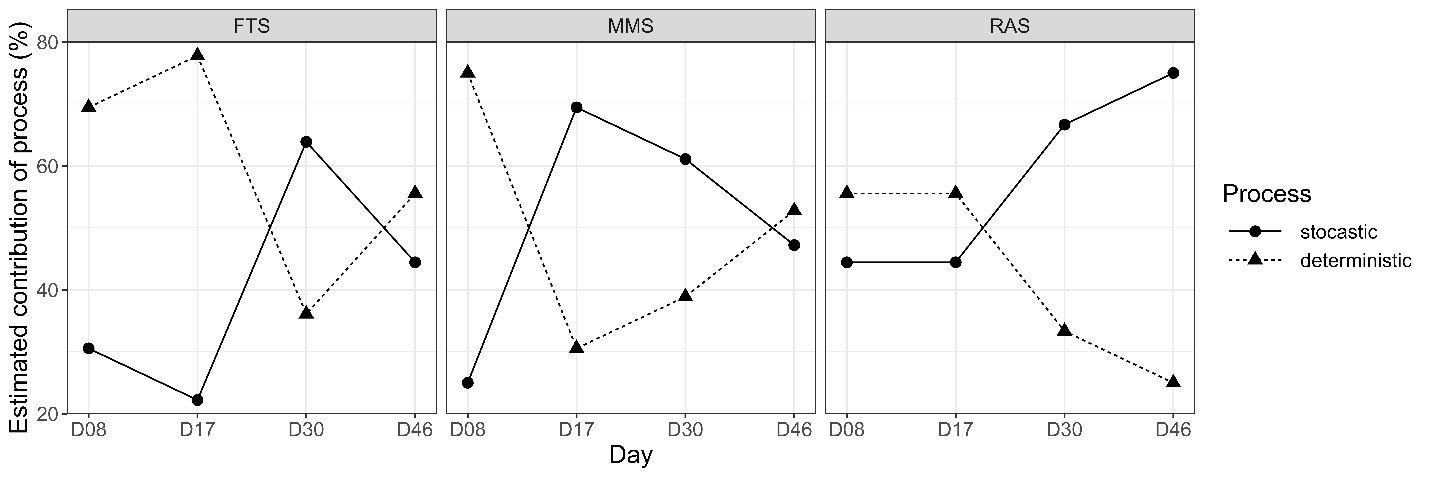


Figure S6: Temporal estimated relative contribution of stochastic and deterministic processes in community assembly for fish samples at each sampling day (D8 – D46) within each treatment system (FTS, MMS and RAS). Estimations are based on β-NTI values of 36 comparisons between individual fish (9 fish per treatment). |β-NTI| < 2 represents comparisons that are not significantly different from the null model and indicate stochastic community assembly and |β-NTI| > 2 indicate deterministic community assembly.


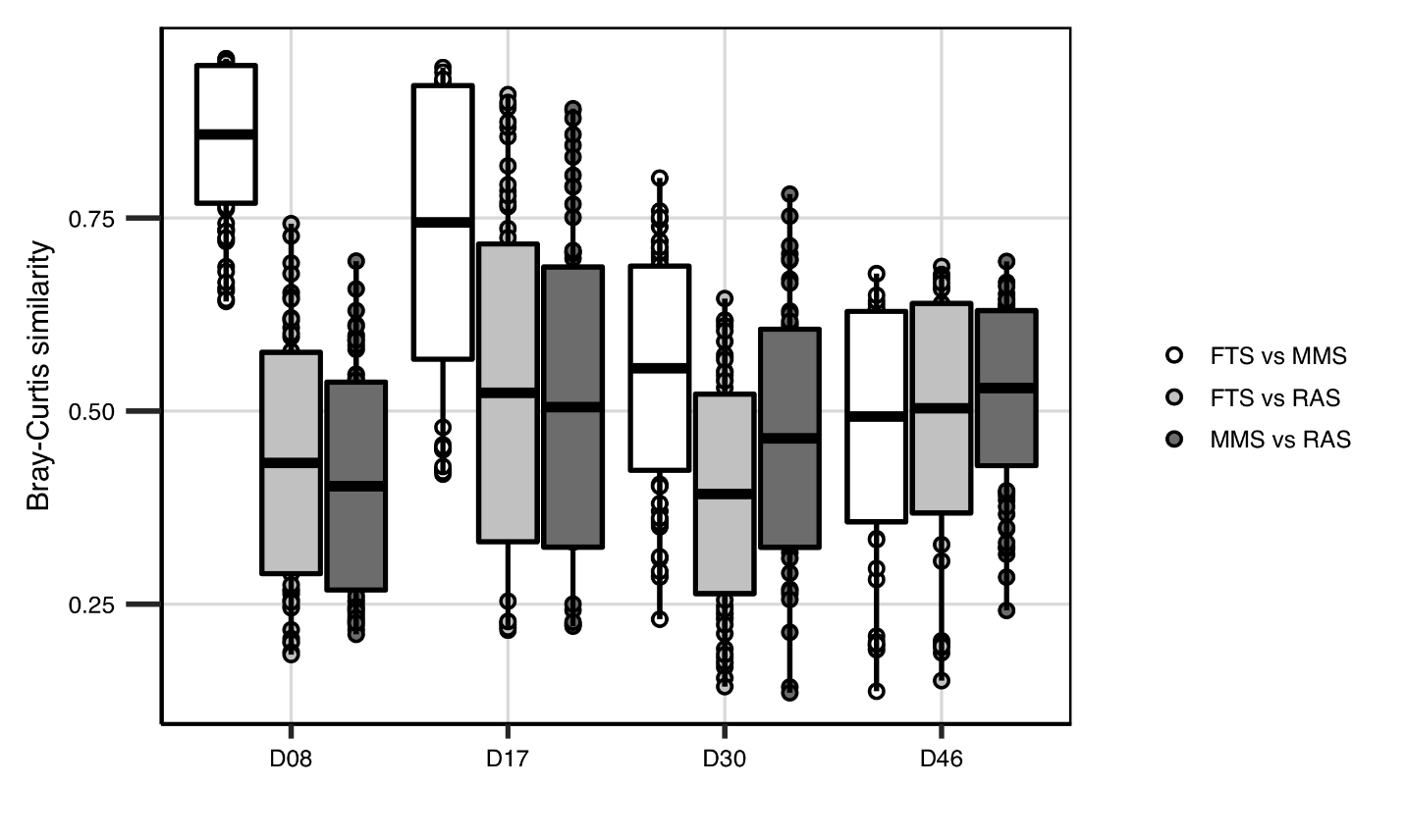


Figure S7: Average Bray-Curtis similarities of the bacterial communities of the cod larvae at 8, 17, 30 and 46 dph (D4 – D46) between systems (FTS, MMS and RAS). Average values are based on comparisons between 9 samples from each system and sampling time. Error bars are standard deviations.


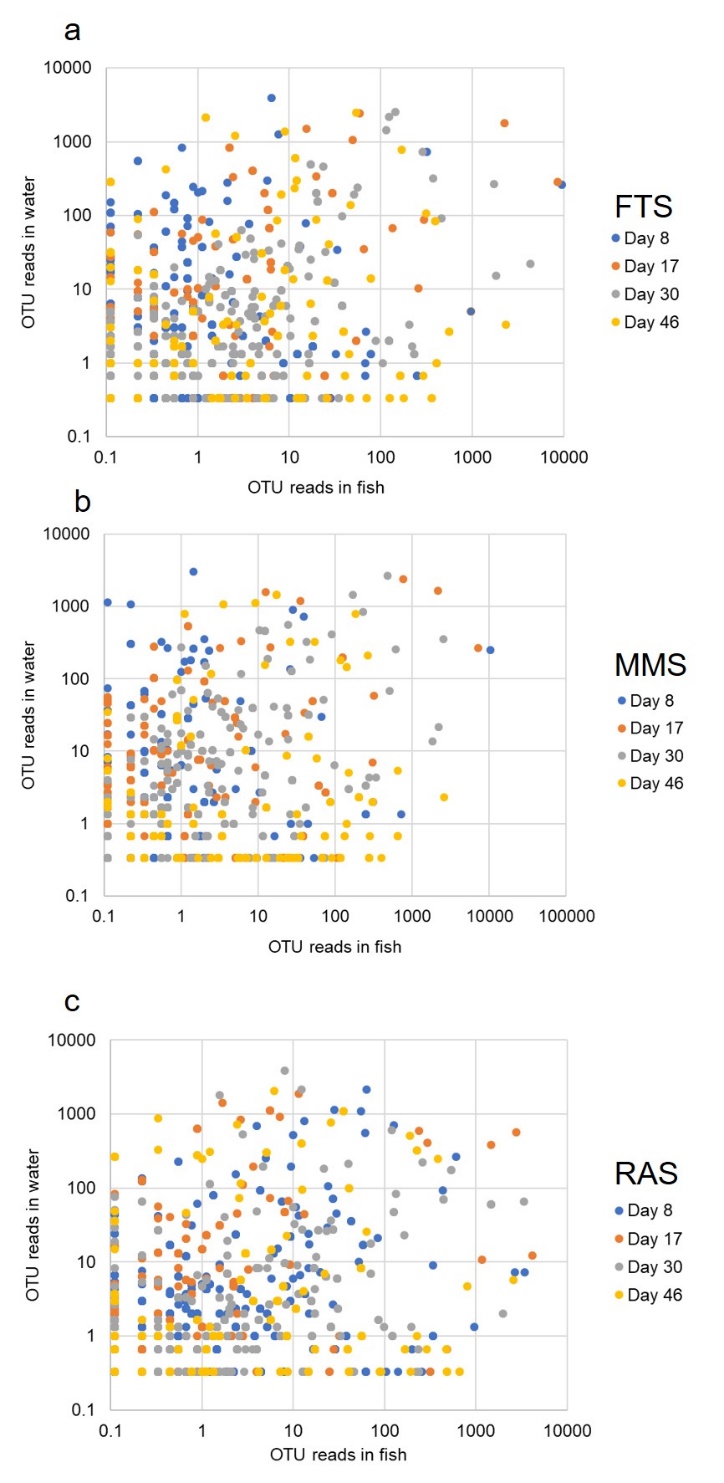


Figure S8: Correlation of the number of reads of each OTU detected in both fish and water samples at 8, 17, 30 and 46 dph (Day 8 – Day 46), in a) FTS, b) MMS and c) RAS.

Table S3: Relative abundances (given as percentages of total reads) for OTUs reaching higher abundances than 2% of the reads for both fish (average of nine) and water (average of three) samples for at least one system at one sampling time.

|  | OTU_1  *Arcobacter* | | OTU_3  *Marinomonas* | | OTU_5  *Pseudoalteromonas* | | OTU_7  *Microbacterium** | | OTU_13  *Aliivibrio* | |
| --- | --- | --- | --- | --- | --- | --- | --- | --- | --- | --- |
| Sample | Fish | Water | Fish | Water | Fish | Water | Fish | Water | Fish | Water |
| FTS 8 dph | 78.4 | 2.2 | 0.1 | 10.5 | 2.7 | 6.1 | 0.6 | 0.0 | 0.1 | 0.7 |
| FTS 17 dph | 70.7 | 2.3 | 18.7 | 14.9 | 0.4 | 8.7 | 0.0 | 0.0 | 0.5 | 19.8 |
| FTS 30 dph | 35.9 | 0.2 | 3.8 | 0.7 | 1.0 | 17.8 | 14.2 | 2.2 | 2.4 | 6.1 |
| FTS 46 dph | 2.6 | 0.9 | 0.0 | 3.5 | 1.4 | 6.4 | 0.7 | 0.0 | 3.3 | 0.7 |
| MMS 8 dph | 85.2 | 2.1 | 0.2 | 7.4 | 0.3 | 6.0 | 0.1 | 0.0 | 0.0 | 0.4 |
| MMS 17 dph | 58.8 | 2.2 | 17.8 | 13.7 | 0.3 | 9.9 | 0.0 | 0.0 | 6.3 | 19.9 |
| MMS 30 dph | 15.2 | 0.1 | 4.3 | 0.6 | 3.9 | 22.0 | 20.8 | 2.9 | 1.9 | 6.9 |
| MMS 46 dph | 2.2 | 1.8 | 1.0 | 1.5 | 1.5 | 6.5 | 1.6 | 0.0 | 1.2 | 1.2 |
| RAS 8 dph | 28.3 | 0.1 | 1.0 | 5.8 | 5.0 | 2.2 | 0.4 | 0.1 | 0.2 | 0.4 |
| RAS 17 dph | 34.8 | 0.1 | 22.9 | 4.7 | 2.5 | 3.4 | 2.6 | 0.0 | 12.2 | 3.2 |
| RAS 30 dph | 1.4 | 0.2 | 1.1 | 0.7 | 1.0 | 5.0 | 28.1 | 0.5 | 2.2 | 1.8 |
| RAS 46 dph | 1.6 | 4.2 | 0.0 | 1.0 | 1.9 | 2.6 | 0.3 | 0.8 | 3.2 | 2.1 |

*As classified by RDP Classifier and SILVA

Table S4: The 5 OTUs that contribute most to the differences in bacteria between fish in RAS and FTS/MMS (SIMPER analysis), at 8, 17 and 30 dph, and average % of reads in corresponding water samples.

| **8 dph** | | | | | | | |
| --- | --- | --- | --- | --- | --- | --- | --- |
| **OTU ID** | **Contrib. %** | **Mean FTS/MMS fish** | **average % of reads FTS/MMS fish** | **Mean RAS fish** | **average % of reads RAS fish** | **average % of reads MMS/FTS water** | **average % of reads RAS water** |
| OTU_1 | 45.98 | 9900 | 81.8 | 3420 | 28.3 | 2.1 | 0.058 |
| OTU_4 | 13.06 | 850 | 7.0 | 2670 | 22.1 | 0.026 | 0.060 |
| OTU_16 | 5.13 | 249 | 2.1 | 965 | 8.0 | 0.0083 | 0.011 |
| OTU_5 | 5.04 | 180 | 1.5 | 607 | 5.0 | 6.0 | 2.2 |
| OTU_25 | 3.08 | 1.56 | 0.012 | 433 | 3.6 | 0.031 | 0.77 |
| **17 dph** | | | | | | | |
| **OTU ID** | **Contrib. %** | **Mean FTS/MMS fish** | **average % of reads FTS/MMS fish** | **Mean RAS fish** | **average % of reads RAS fish** | **average % of reads MMS/FTS water** | **average % of reads RAS water** |
| OTU_1 | 32.88 | 7840 | 64.8 | 4210 | 34.8 | 2.3 | 0.050 |
| OTU_3 | 17.28 | 2210 | 18.3 | 2770 | 22.9 | 14.3 | 2.6 |
| OTU_13 | 14.05 | 408 | 3.4 | 1480 | 12.2 | 19.9 | 1.8 |
| OTU_22 | 9.87 | 52.8 | 0.44 | 1180 | 9.8 | 0.29 | 0.041 |
| OTU_7 | 2.70 | 2.44 | 0.020 | 316 | 2.6 | 0.023 | 0.20 |
| **30 dph** | | | | | | | |
| **OTU ID** | **Contrib. %** | **Mean FTS/MMS fish** | **average % of reads FTS/MMS fish** | **Mean RAS fish** | **average % of reads RAS fish** | **average % of reads MMS/FTS water** | **average % of reads RAS water** |
| OTU_1 | 21.31 | 3090 | 25.5 | 166 | 1.4 | 0.15 | 0.19 |
| OTU_7 | 13.28 | 2120 | 17.5 | 3400 | 28.1 | 2.6 | 0.55 |
| OTU_12 | 10.46 | 20.8 | 0.17 | 1460 | 12.1 | 4.3 | 0.50 |
| OTU_4 | 6.12 | 2020 | 16.7 | 1970 | 16.3 | 0.15 | 0.017 |
| OTU_34 | 3.50 | 491 | 4.06 | 8.33 | 0.069 | 2.4 | 0.022 |
